# Climatological predictions of the auroral zone locations driven by moderate and severe space weather events

**DOI:** 10.1038/s41598-022-25704-2

**Published:** 2023-01-15

**Authors:** Stefano Maffei, Joseph W. B. Eggington, Philip W. Livermore, Jonathan E. Mound, Sabrina Sanchez, Jonathan P. Eastwood, Mervyn P. Freeman

**Affiliations:** 1 Earth and Planetary Magnetism Group, Institute of Geophysics, ETH Zurich, Zürich, Switzerland; 2 School of Earth and Environment, University of Leeds, Leeds, UK; 3 Blackett Laboratory, Space and Atmospheric Physics Group, Imperial College London, London, UK; 4 Institut de Physique du Globe de Paris, Université Paris-Diderot, Paris, France; 5 British Antarctic Survey, High Cross, Madingley Road, Cambridge, UK

**Keywords:** Aurora, Core processes

## Abstract

Auroral zones are regions where, in an average sense, aurorae due to solar activity are most likely spotted. Their shape and, similarly, the geographical locations most vulnerable to extreme space weather events (which we term ‘danger zones’) are modulated by Earth’s time-dependent internal magnetic field whose structure changes on yearly to decadal timescales. Strategies for mitigating ground-based space weather impacts over the next few decades can benefit from accurate forecasts of this evolution. Existing auroral zone forecasts use simplified assumptions of geomagnetic field variations. By harnessing the capability of modern geomagnetic field forecasts based on the dynamics of Earth’s core we estimate the evolution of the auroral zones and of the danger zones over the next 50 years. Our results predict that space-weather related risk will not change significantly in Europe, Australia and New Zealand. Mid-to-high latitude cities such as Edinburgh, Copenhagen and Dunedin will remain in high-risk regions. However, northward change of the auroral and danger zones over North America will likely cause urban centres such as Edmonton and Labrador City to be exposed by 2070 to the potential impact of severe solar activity.

## Introduction

Aurorae are the best known visual expression of space weather activity. They are caused by ionisation in the upper atmosphere by charged magnetospheric particles, accelerated during periods of enhanced geomagnetic activity. These charged particles travel along magnetic field lines and because of the primarily dipolar nature of the geomagnetic field, aurorae are preferentially observed at high latitudes in approximately oval-shaped regions (the auroral ovals) surrounding the geomagnetic poles (the intersection points of the geomagnetic dipole axis and the surface of Earth). The auroral ovals are important features of the magnetospheric response to space weather phenomena, as they are associated with increased electromagnetic disturbances, visible in both ground- and satellite-based observations^1,2^, and particle precipitation that has degrading effects on satellite technology^3,4^. Accurate determination of the future locations where, on average, aurorae sightings are most likely in the future also has consequences for the tourism industry and aurora-watching trip planning.

The exact shape of the auroral ovals is controlled by the instantaneous position of Earth with respect to the Sun, the physical conditions of the solar wind and the configuration of the geomagnetic field. Over longer timescales, e.g. longer than the 11 years of a solar cycle, the auroral ovals can be approximated by the auroral zones, which, for low-to-moderate solar activity, are traditionally represented by quasi-circular belts centered around the geomagnetic poles and located between 65 and 70$^\circ$ of geomagnetic latitude^5–7^. Using this definition, the auroral zone location and shape do not depend on instantaneous solar wind conditions and their temporal evolution can be solely linked to the changes in the geomagnetic field. This allows us to consider space climate, a moving average of space weather conditions over the decadal timescales dictated by the evolution of the geomagnetic field of internal origin. Previous studies estimated the past evolution of these low-solar-activity auroral zones^2,8–10^ or of their poleward edges alone (encircling the so-called polar caps)^11^ and found significantly different shapes and temporal changes for the Northern and Southern zones. In particular, since the beginning of the 20th century, the evolution of the Northern auroral zone can be essentially described by a drift from North America towards Siberia^2,8,10,11^, in qualitative agreement with the recent rapid motions of the North Magnetic pole^12–17^. At the same time, the Southern auroral zone evolution is better described by an elongation towards the equator in the Atlantic Hemisphere^8,10,11^. The auroral zones and polar cap surface areas have been decreasing since 1940^11^ for the Northern Hemisphere and increasing since at least the beginning of the 20th century for the Southern Hemisphere. This asymmetric behaviour is at odds with expected scaling laws valid under the assumption of a dipole-dominated main field^18–20^, according to which both polar cap areas should increase as the dipole intensity decayed^21–23^.^11^ suggested that the shrinking of the northern auroral zone is due to a local strengthening of the high-latitude geomagnetic field intensity in the Northern Hemisphere, contrary to the geomagnetic field evolution in the Southern Hemisphere. Similarly, the recent rapid drift of the North Magnetic Pole can be explained by the weakening (strengthening) of the patch of high magnetic field intensity above Canada (Siberia) and the concurrent drift towards Siberia of the whole magnetic pattern^17^.

Under the influence of severe solar activity, the auroral oval is pushed to lower latitudes and the electromagnetic disturbances associated with it increase in magnitude, with damaging effects on ground-based technology^24^. In extreme cases, such as during the 1859 Carrington event, aurorae and electromagnetic disturbances were detected as far south as 10 degrees latitude^25^. Using extreme event statistics informed by available ground-based geomagnetic field observations,^26^ estimated that the largest geomagnetic field perturbations for extreme space weather events (100 and 200 years return period), can be expected in a range between geomagnetic centered-dipole latitudes^27,28^ of about $50^\circ$ and $60^\circ$, which we term the ‘danger zone’. Presently, these geomagnetic latitudes span a large part of Northern Europe and Russia, the Northern United States and Southern Canada, the island of Tasmania and Southern New Zealand. These are the locations most exposed to the risk of geomagnetically-induced currents (GIC) driven by severe space weather events. As severe GIC could cause significant damage to power grids, rail networks and communication systems, predicting the location of their most intense occurrences aids the crafting of space weather mitigation strategies across the globe.

Predictions of the future location of the auroral and danger zones can help inform industry and policy makers, and feed into the timely development of effective space weather mitigation strategies. To our knowledge, the only auroral zone forecast available in the literature is presented in^29^, in which a simple description for the centennial evolution of the geomagnetic field coefficients (the Gauss coefficients, see Methods) is extrapolated over a 1000 years temporal window. However, new physics-based forecasts are available, which model the expected dynamics within the core^30,31^ to predict the evolution of the internally generated geomagnetic field over decadal timescales. We will use these to produce new and likely much better forecasts of the auroral zone shape and location.

## Results

### Description of the auroral and danger zones

**Figure 1:**
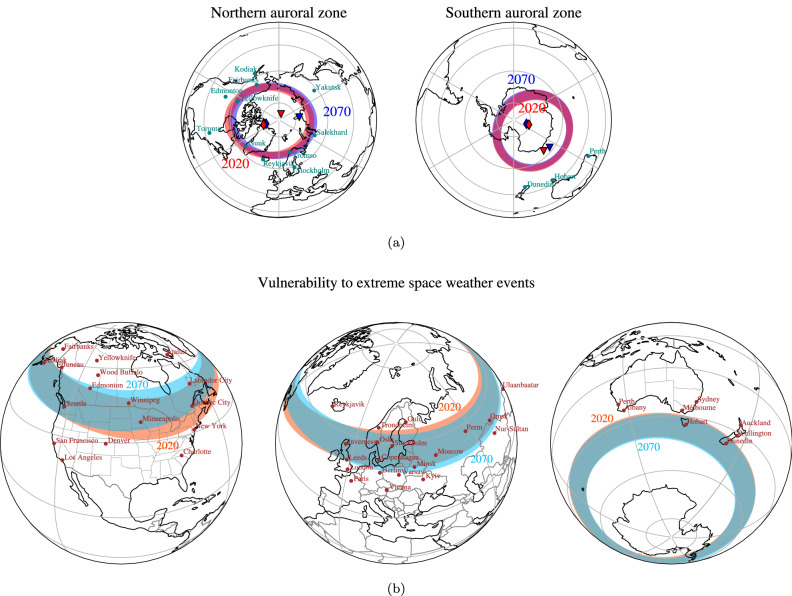
Auroral zones and danger zones for 2020 and 2070 for the mean MPG forecast. (**a**) Auroral zones for the 2020 (in red) and 2070 (in blue) epochs. The AACGM latitudes for the 2020 epoch are calculated from geomagnetic field coefficients given by the IGRF-13. For the 2070 epoch the AACGM coordinates were calculated from the mean MPG forecast. The red and blue diamonds indicate the location of the geomagnetic poles at 2020 and 2070, respectively. The red and blue triangles indicate the location of the magnetic dip poles at 2020 and 2070, respectively. (**b**) Near-side projection of the regions exposed to severe space weather events (‘danger zones’) for the 2020 (the orange bands) and 2070 (the cyan bands) epochs. The AACGM latitudes for the 2020 epoch are calculated from geomagnetic field coefficients given by the IGRF-13. For the 2070 epoch the AACGM coordinates were calculated from the mean MPG forecast. All maps were generated in Python 3.8 with the library cartopy (version 0.18.0).

We consider the auroral zones as a climatological average of the auroral ovals for low-to-moderate solar wind conditions, which are defined by the latitudinal bands between 65 and $70^\circ$ in Altitude Adjusted Corrected Geomagnetic (AACGM) coordinates (see Methods). This choice is motivated by interannual averages of the auroral oval boundaries based on satellite images^32^ and has been corroborated by complementary magnetospheric simulations with the global magnetohydrodynamic code Gorgon, for various choices of interplanetary magnetic field (IMF) intensity (see Methods). We find that for a wide range of IMF strengths typical of low-to-moderate solar wind conditions, the poleward bound of the auroral zones can indeed be effectively described by a geomagnetic latitude of $70^\circ$ (see Supplementary Fig. S1). Analogous methodologies allow us to quantify the climatological exposure to high-risk space weather events by following the temporal evolution of the danger zones, the $50^\circ$-to-$60^\circ$ geomagnetic latitude band in AACGM coordinates (see Methods). For auroral zones and danger zones from 1900 to 2020 we consider the geomagnetic field to be described by the IGRF-13 field model^23^. Note that the conversion between AACGM and geographic coordinates depends on the full set of Gauss coefficients describing the geomagnetic field and its temporal evolution. The auroral and danger zones for the year 2020 are illustrated in Fig. 1.

Due to the non-dipolar contributions to the geomagnetic field, the northern and southern zones have different shapes that depart significantly from circular rings. The boundaries of the northern zone appear similar to ellipses elongated towards North-America and Siberia, while the southern zone is significantly compressed in its South-Atlantic sector. The shape of the 2020 auroral zones is consistent with previous descriptions of the auroral zones over the 20th and 21st centuries^2,8–11,29^.

**Figure 2:**
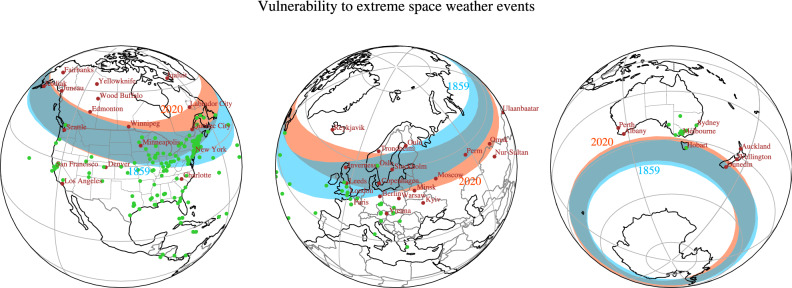
High-exposure regions to severe space weather events (‘danger zones’) for 2020 and 1859, the year of the Carrington Event. $50^\circ$-to-$60^\circ$ AACGM latitudinal bands for 2020 (the orange bands) and 1859 (the cyan bands), shown via a near-side projection. The geomagnetic field for 1859 and 2020 is described by the coefficients of the *gufm1* and IGRF-13 models, respectivley. Locations of selected cities inside or in the proximity of the danger zones are also reported. The green dots indicate sightings of auroral activity reported between 28th of August and 4th of September, 1859^25^. These maps were generated in Python 3.8 with the library cartopy (version 0.18.0).

In contrast to the auroral zones, the regions that we here term danger zones cannot be rigorously defined in terms of long-term averages of observed quantities. We defined them based on extreme events with estimated return period of 200 years (see Methods and^26^). For such events, we do not have access to a wide enough set of of reliable GIC observations upon which the climatological meaning of the danger zones can be justified. We can, however, corroborate the danger zone definition by comparing its location with electromagnetic disturbances reported during severe events for which written records exist. Furthermore note that the bounds reported in^26^ (see Methods) are expressed in geomagnetic, dipole-centered coordinates, which only depend on the inclination of the geomagnetic dipole. It is therefore necessary to test whether the generalisation to AACGM coordinates is appropriate. The most severe geomagnetic storm in recent history was the 1859 Carrington event^33^; in Fig. 2 we plot aurorae sightings during the event^25^ together with our reconstruction of the danger zones in 1859. For this epoch, we used the *gufm1* model to describe the magnetic field coefficients^34^. Other models for the 1859 are available, such as the COV_OBSx.2 field model^35^. The danger zones locations reported in Fig. 2 are visually identical to those obtained for the COV_OBSx.2 model and we do not replot them here.

The poleward edge of the northern danger zone agrees well with the location of the northernmost aurorae sightings, with the exception of a few aurorae sightings reported in Newfoundland and north of Quebec City. This suggests that the poleward edges of the danger zones are a good measure of the most poleward locations that are most exposed to severe space weather events. In contrast, aurorae sightings during the Carrington event have been reported in regions far more equatorward than the $50^\circ$ AACGM latitude marking the equatorward edge of the danger zones. This can be due to details of the Carrington event not captured by the climatological nature of our study, but it has to be noted that some of the mid-to-low latitude events could be equatorial aurorae^36^, associated with a phenomenology that is not included in our danger zone definition.

### Forecasting the auroral and danger zones

To describe the geomagnetic field for 2020 onward we principally use a state-of-the-art, data-assimilation-based forecast, that we refer to as the ‘MPG forecast’^31^ (see Methods). The MPG forecast describes the evolution of the geomagnetic field over the entirety of the current century. Its ensemble nature allows us to estimate auroral and danger zone location uncertainty (see below). To our knowledge, the only other available physics-based prediction of the geomagnetic field over the same period has been presented in^30^. This prediction, that we refer to as the ‘IPGP forecast’, is produced by a geodynamo simulation initialised from a state given by a single-epoch inversion of geomagnetic observations in 2015. For comparison, we also consider a non-physical, IGRF-based forecast for which the IGRF-13 model predictions for the 2020-2025 coefficients are extrapolated to future epochs (see Methods). As suggested by Gauss coefficients evolution (see Supplementary Fig. S2) the MPG ensemble spread encompasses most of the temporal variability of the IGRF and IPGP forecasts and so provides a sound basis for our auroral forecasts.

Figure 1a shows the temporal changes of auroral zones between the 2020 and 2070 epochs, based on the mean MPG forecast. The temporal horizon of 50 years has been chosen as being short enough for data-assimilation geomagnetic field forecasts to remain accurate^37,38^, and long enough to develop new space weather mitigation policies if needed based on the results presented in this paper. The mean MPG forecast predicts a drift of the northern zone away from North America and towards Siberia. A similar drift can be seen in the location of the North Geomagnetic Pole (the diamonds in Fig. 1a), although its drift direction is not fully correlated with that of the auroral zone. The 2070 southern zone shows very little drift but some elongation in the direction of the Atlantic Hemisphere (towards South-America, the Atlantic Ocean and Africa) and a small net north-westward drift. These results suggest that it will be increasingly unlikely to spot aurorae in North-American locations with the same latitudes as Edmonton and Kodiak, while it will be increasingly likely for locations such as Yakutsk, in Russia. Little change is expected for Europe, Australia and New Zealand. The qualitatively different behaviour in the two hemispheres can only be caused by the non-dipolar components of the main field, since the dipolar field would cause symmetric evolution of both auroral zones (as is the case for the geomagnetic poles). Note that neither the magnetic dip poles (the triangles in Fig. 1a), nor the geomagnetic poles are a satisfactory proxy for auroral zone evolution, as pointed out in^10,11^. This confirms that non-dipolar magnetic field components are important in defining the locations of the auroral zones.

Figure 1b shows the temporal changes of the danger zones between the 2020 and 2070 epochs: the predicted evolution is qualitatively similar to the evolution of the auroral zones (see Fig. 1a). In particular, there is little change in Northern Europe, with the Northern UK, Denmark and Scandinavia still remaining in these high-risk regions in 2070. Most notably, the high-risk band in North America moves northward, resulting in its southern edge shifting from approximately New York City in 2020 to Toronto in 2070. Concerning the southern high-risk bands, the largest predicted change is a motion towards South America in the Southern Atlantic Ocean. In the Indian and Pacific Ocean the changes are modest, with a small drift southward. In particular, the island of Tasmania and Southern New Zealand (including the city of Dunedin), will remain in the same risk region between 2020 and 2070, according to our forecast.

For all forecasts considered here, the qualitative evolution of both auroral and danger zones is similar but different in magnitude (see Supplementary Fig. S3). As for the Gauss coefficients, the IPGP and IGRF predictions are contained within the ensemble spread of the MPG forecast. The uncertainty for the 2070 zone boundaries can be estimated to be about $2^\circ$ for all longitudes except for the Atlantic sector of the southern zones, for which the uncertainty is about $5^\circ$.

In accordance with previous work^11,17^, we can identify features in the geomagnetic field evolution that correlate with the future evolution of the auroral and danger zones predicted here. In the Northern Hemisphere, all forecasts considered in this study (the MPG, IPGP and IGRF forecasts) predict that the recent evolution of the high-intensity patches will continue over the next 5 decades, with the Canadian patch weakening and drifting towards Siberia, while the Siberian patch strengthens (see Supplementary Fig. S4). In the Southern Hemisphere, the high-intensity patch located between Antarctica and Australia is predicted to drift westward, which visually correlates with the evolution of the southern auroral and danger zones (see Fig. 1a). Although interpreting the evolution of the auroral and danger zones in terms of high-latitude geomagnetic field variations appears physically reasonable, we note that no study to date quantitatively links the evolution of the two; indeed, a contribution from low-latitude geomagnetic features cannot yet be excluded. A subsequent study will make use of a more quantitative approach to explore which geomagnetic field features are responsible for the location and evolution of the auroral and danger zones.

### Forecast for selected cities

**Figure 3:**
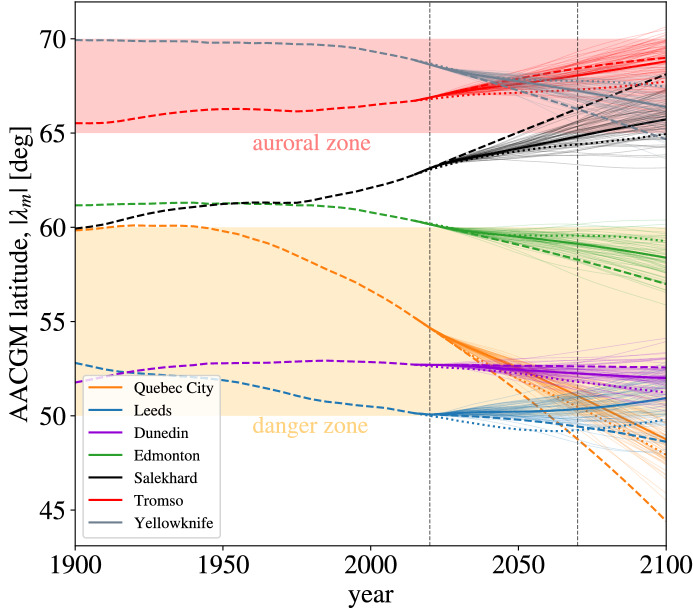
Evolution of the absolute value of AACGM latitude for selected cities. The selected locations are also reported in Fig. 1. The dashed vertical lines indicate the 2020 and the 2070 epochs. The shaded areas indicate the limits of the auroral and ‘danger’ zones ($65^\circ$-to-$70^\circ$ and $50^\circ$-to-$60^\circ$ AACGM latitudes, respectively). The IGRF-13 model (up to 2020) and the IGRF forecast (after 2020) are represented by the dashed lines. The mean MPG forecast and ensemble members are represented by continuous thick and thin lines, respectively. The IPGP forecast is represented by dotted lines.

In Fig. 3 we report the temporal evolution of the absolute value of the AACGM latitude, $\uplambda _m$, for selected cities chosen for their possible exposure to space weather events (Quebec City, Leeds, Dunedin, Edmonton) or for being popular aurora-watching destinations (Dunedin, Edmonton, Salekhard, Tromso, Yellowknife).

From Fig. 3 we can see a more detailed picture of what already indicated in Fig. 1. For example, both Tromso and Yellowknife will remain inside the auroral zone over the next 50 years, and the likelihood of aurora sightings is not expected to vary (on average). By the end of the century, however, some of the forecasts predict that both cities might find themselves at the edge of the auroral zones, and that perhaps aurorae sightings could be less frequent than today. Salekhard, on the other hand, is expected to enter the auroral zone over the next few decades and to then remain in the zone at least until 2100, according to the majority of the forecasts. We can therefore predict that over the next century, the likelihood of spotting aurorae in Salekhard is expected to increase.

Concerning the evolution of the danger zones, from Fig. 3 we see that Quebec City currently has a geomagnetic latitude of $\uplambda _m\simeq 55^\circ$, and that this value is projected to decrease significantly in the next few decades, so that by 2070 Quebec City will be located at the edge of, or outside the danger zone, depending on the forecast. Edmonton is currently placed at the poleward edge of the danger zone and, similar to Quebec City, its AACGM latitude will decrease with time, which in this case suggests increasing exposure to extreme space weather events. The geomagnetic latitudes of the locations in the UK and New Zealand (Leeds and Dunedin) do not show the same amplitude of variations as Quebec City and Edmonton, indicating a small variation in their exposure to severe space weather events. For example, all forecasts predict, for 2070, small variations in the geomagnetic latitude for Leeds (of the order of $2^\circ$ with respect to $\uplambda _m=50^\circ$) and no significant change in terms of risk assessment.

The present study illustrates the applicability of current geomagnetic field forecasts for space climate applications. Our results indicate the need for high awareness to the risk posed by severe space weather events in Canada, an increasingly large area of which we predict will be located within the danger zones over the next 50 years. Continuing efforts are needed in presently at-risk regions for which no significant change is estimated, such as Southern New Zealand and Northern UK. We have also shown (see Fig. 3 and Supplementary Figures S2 and S3) that the forecast uncertainty related to the spread of MPG forecast’s ensemble members is comparable to the uncertainty arising from differences between forecasts (here, the IPGP and IGRF forecasts). Therefore inter-model forecast accuracy is a good estimate of intra-model accuracy. As more multi-decadal geomagnetic field forecasts become available this observation will be crucial in building confidence in their reliability.

## Methods

### Definition of auroral zones and regions of severe space weather exposure

We identify the auroral zones under low-to-moderate solar wind conditions (simply, the ‘auroral zones’) by the geomagnetic latitudinal bands between 65 and $70^\circ$ in Altitude Adjusted Corrected Geomagnetic (AACGM) coordinates^28,39,40^. Our definition of the auroral zones is similar to that employed in previous studies^2,8,9,29^ where typically the Magnetic Apex coordinates system is used instead^28,41,42^. The choice of $65^\circ$ and $70^\circ$ as the geomagnetic latitudinal bounds for the auroral zones can be justified by the temporal averages of aurorae occurrences as observed from the IMAGE satellite between 2000 and 2005^32^ and it is corroborated by the analysis of multi-decadal geomagnetic field measurements from high-latitude stations^2^. Furthermore, following the results of^26^, we define the regions most exposed to severe space weather events (and hereby named ‘danger zones’) as the geographical regions of Earth’s surface where the highest geomagnetically induced currents (GIC) are expected to be driven by temporal geomagnetic variations. For catastrophic space weather events with 200-years return period,^26^ estimated that the auroral zones can be defined by the geomagnetic latitudinal band between 50 and $60^\circ$, with peak geomagnetically induced currents (GIC) being expected at about $55^\circ$ (see Figure 6 of^26^). We therefore define the danger zones as being bound by AACGM latitudes $50^\circ$ and $60^\circ$.

In order to calculate AACGM latitude, $\uplambda _{m}$, we make use of the freely available Python package aacgmv2 version 2.6.2 (https://github.com/aburrell/aacgmv2, see also^40^), which allows conversion between geographical and AACGM coordinates in accordance to a given geomagnetic field model, for a specified epoch and a given altitude above Earth’s surface. Since our focus is on the variations in geomagnetic latitude caused by decadal changes in the internal geomagnetic field, the epochs for which AACGM coordinates are calculated are defined by the year alone and we fix the day of the year to be the first of January. Considering a different day of the year would not alter the results presented here.

AACGM coordinates are used to calculate geomagnetic latitude and longitude of a given geographic location making use of a full description of the geomagnetic field, including its non-dipolar components. The AACGM coordinates corresponding to a given geographic location at the surface of Earth are calculated by following the geomagnetic field line from the geographic location to the magnetic dipole equator, then following back to the surface of Earth along the dipole field line intersecting the point on the magnetic equator. The AACGM coordinates are then given by the latitude and longitude of the intersection of the dipole field line with the surface of Earth. Because all points along a magnetic field line have the same geomagnetic latitude and longitude, AACGM coordinates are widely used in magnetospheric studies since they allow researchers to naturally track particle precipitation along these lines. By construction, AACGM coordinates are not well defined in an equatorial region making this coordinate system unreliable for equatorial and low-latitudes application. For high-latitude applications (i.e., at latitude greater than about $50^\circ$), however, the AACGM coordinate system is in excellent agreement with, for example, Magnetic Apex and Quasi-Dipole coordinates^28^. The advantage of AACGM coordinates over Magnetic Apex coordinates is the availability of fast routines to calculate the conversion from and to geographic coordinates. For more details on the AACGM coordinate system, the reader is invited to consult^28,39,40^ and references therein.

### Comparison of auroral zone boundaries to global magnetosphere simulations

To further place into context our definition of typical auroral zone latitudes, we perform a comparison to predictions from a global magnetohydrodynamic (MHD) model, Gorgon(^43,44^), with which we simulate the coupled magnetosphere-ionosphere system under different strengths of solar wind driving. We employ a high resolution (0.25 $r_e$, where $r_e=6371$ km is the mean radius of Earth) regular cartesian grid^45^, with domain spanning X = (−30, 90) $r_e$, Y = (−40, 40) $r_e$, and Z = (−40, 40) $r_e$, related to GSM coordinates by (X, Y, Z) = (−X$_{GSM}$, −Y$_{GSM}$, Z$_{GSM}$). The inner boundary is placed at 3 $r_e$, where we map field-aligned currents to a thin-shell ionosphere model which sets the plasma flow as an inner boundary condition^46^. A dipole magnetic field is placed at the origin, with moment $M = 7.71\times 10^{22}$ Am$^2$ as per the IGRF-13 model for 2020. The dipole tilt angle (that between the dipole axis and the GSM Z-axis) is set to zero as we assume an equinox configuration, effectively averaging out diurnal and seasonal variations.

The solar wind has steady synthetic conditions of velocity $v_x$ = 400 kms$^{-1}$, number density *n* = 5 cm$^{-3}$ and ion/electron temperatures $T_{i,e}$ = 5 eV, consistent with a slow, ambient solar wind. We simulate separately with southward IMF of increasing magnitude, $B_z$ = −2 nT, −4 nT, −6 nT, −8 nT, and −10 nT, as well as the more severe case of −20 nT. It should be stressed that the solar wind speed also modulates the strength of coupling with the magnetosphere, but we keep this (and the other parameters) constant to simplify any comparison. A sense of the strength of driving with relation to geomagnetic activity can be inferred by calculating the power input into the magnetosphere, as approximated by the $\epsilon$ parameter^47^:

1 $$\begin{aligned} \epsilon = \frac{4\pi }{\mu _0}vB^2\sin ^4\left( \theta _{IMF}/2\right) l_0^2. \end{aligned}$$

Here $\theta _{IMF} = \arctan (B_y/B_z)$ is the IMF clock angle, equal to 180$^\circ$ in our purely southward IMF case, and $l_0$ is the length scale of the solar wind coupling, typically taken as equal to 7 $r_e$ as we assume here. A power input exceeding about $10^{11}$ W is associated with the occurrence of substorms, whilst an extended period with power input above $10^{12}$ W can drive geomagnetic storms(^48,49^). Using the values for our simulated solar wind conditions we would thus expect substorm activity (and enhanced space weather risk) for IMF strengths of $\sim$4 nT (for which $\epsilon = 1.3\times 10^{11}$ W) and above, with weak storms occurring for IMF strengths above $\sim$10 nT ($\epsilon = 0.8\times 10^{12}$ W). Our range of values between −2 and −10 nT thus encapsulates low-to-moderate space weather conditions. In contrast for the 20 nT case we have $\epsilon = 3.2\times 10^{12}$ W, representing a fairly strong storm for which the auroral oval may be significantly displaced to lower latitudes. Values of $\epsilon$ much greater than this can be considered quite extreme and should result in auroral oval latitudes far from ‘typical’, instead venturing closer towards our ‘danger zone’ latitudes. Therefore from the chosen range of IMF strengths we can identify the polar cap size for different (but not extreme) levels of geomagnetic activity, and hence under what conditions our zone definitions are applicable.

The magnetosphere is initialised up to 2 h with each set of driving conditions, such that it enters a quasi-steady state. Note that we use a uniform ionospheric Pedersen conductance of 10 mho and zero Hall conductance; whilst this does not consider ionisation effects such as auroral precipitation, we are only interested in identifying the average location of the auroral zone rather than exploring any local time-dependence or more complex ionospheric electrodynamics. After 2 h of simulation time we locate the open-closed field line boundary (OCB) which encloses the ionospheric polar cap, corresponding to the poleward boundary of the auroral oval^50^. The OCB is identified by sampling magnetic connectivity near the inner boundary (see^51^). We then map the OCB coordinates down along dipole field lines to the ground at a radius of 1 $r_e$, and calculate the mean and standard deviation of the polar cap latitude $\uplambda _{pc}$ in each case, providing an estimate of the spatial range of the OCB for each IMF strength for all magnetic local times. The result is a series of circular polar caps with constant centered-dipole latitude, representing the approximate poleward boundary of the auroral zone which expands to lower geomagnetic latitudes with increasing IMF magnitude.

We show the resulting polar caps in Supplementary Fig. S1a, visualised in geographic coordinates by centering the circles about the 2020 IGRF-13 North geomagnetic pole. The polar cap expansion (growth in the size of each oval) appears more sensitive to an increase in IMF strength when the IMF is weaker, whilst the polar cap sits on average only $\sim 2.5^\circ$ lower in latitude for −20 nT than for −10 nT. This is demonstrated in Supplementary Fig. S1b, which also compares $\uplambda _{pc}$ to the defined auroral zone of $65^\circ< \uplambda _m < 70^\circ$, neglecting any small differences between geomagnetic latitudes and local-time-averaged AACGM latitudes (which reduce to the former when Gauss coefficients above degree 1 are ignored). For IMF strengths from −6 to −10 nT, i.e. moderate space weather conditions, the mean latitude lies well within the typical auroral zone defined in this study, and even for the weakest IMF strengths some portion of the auroral oval (which will extend a few degrees below $\uplambda _{pc}$) will intersect with this region. Note that the error bars capture the effects of the displacement of the oval from the pole, being more apparent for weaker IMF hence a larger spread, as well as local perturbations along the OCB, which are larger for stronger IMF due to more time-dependent nightside magnetic reconnection.

For $B_z \lesssim$ −20 nT, however, the majority of the auroral oval will lie outside of the defined auroral zone. This is to be expected since the −20 nT case represents more intense space weather conditions (capable of generating a disturbance storm time index below even −200 nT dependent on solar wind speed, see^52^). However these latitudes are still poleward of our defined danger zone of $50^\circ<\uplambda _m<60^\circ$, indicating that even stronger driving conditions are required to achieve this. Indeed, previous simulations of a ‘Carrington-type’ CME, i.e. a centennial timescale event, have predicted OCB geomagnetic latitudes as low as $\sim$ 40$^\circ$^53^. This would correspond to much more extreme upstream solar wind conditions than used in our runs (e.g., the above study assumed $v_x$ $\sim$ 2000 km s$^{-1}$ and IMF $B_z$ $\sim$ −200 nT). However simulating an event of this type requires a specially-tailored and computationally expensive model setup capable of dealing with the extremely compressed state of the magnetosphere, and so is outside the scope of the present study. Nonetheless, our simulation results indicate the chosen range of typical auroral zone latitudes is appropriate for a wide range of driving conditions up to and including weaker geomagnetic storms, and that our danger zone latitudes based on the results of^26^ are consistent only with extreme space weather events.

One final point to consider which we have not discussed above is the sensitivity of the polar cap size (and hence auroral zone) to the strength of the geomagnetic dipole; arguments based on changes in the size of the magnetosphere yield a theoretical dependence of $\cos (\uplambda _{pc}) \propto M^{-1/6}$ (^18,54^). The reason for this is that a constant reconnection voltage (i.e. rate of magnetic flux opening) across the dayside magnetopause will generate a fixed amount of open flux in the steady-state magnetosphere. For a weaker (stronger) dipole and given the same external solar wind driver, this would result in a larger (smaller) polar cap which encloses the same magnetic flux. However since $\uplambda _{pc}$ scales only weakly with the dipole moment, the decrease of $\sim$ 2% in *M* predicted by the 2070 MPG forecast ($M = 7.57\times 10^{22}$ Am$^2$) would result in a very small increase of $\sim 0.1^\circ$ in $\uplambda _{pc}$. Indeed, in internal investigations where we repeated our simulations with the 2070 MPG dipole intensity we found only negligible differences in OCB latitudes. Thus we expect morphological differences in the auroral and danger zones arising from the internal field evolution to dominate over changes in its overall size for the forecast scenarios investigated here, such that the fixed definition of zone latitudes is valid.

### Geomagnetic field models and forecasts

As input to the aacgmv2 algorithm, a geomagnetic field model is required. As is commonplace^55^, we describe the evolution of the geomagnetic field, ${\mathbf {B}}$, above Earth’s surface via its Gauss coefficients $g_l^m$ , $h_l^m$ and the following spherical harmonics decomposition:

2 $$\begin{aligned} {{\textbf {B}}} = -r_e \nabla \left[ \sum _{l=1}^{L}\sum _{m=0}^l\left( \frac{r_e}{r}\right) ^{l+1} \left[ g_l^m\cos (m\phi ) + h_l^m \sin (m\phi ) \right] P_l^m(\cos \theta )\right] , \end{aligned}$$

where $r_e=6371$ km is the mean radius of Earth, $(r,\theta ,\phi )$ are the geographic spherical coordinates with origin in the center of Earth (hereafter considered spherical for simplicity), $P_l^m(x)$ are the associated Legendre functions of degree *l* and order *m*, *L* is the maximum degree of expansion and $\nabla$ is the gradient operator acting on the spatial coordinates. To describe the geomagnetic field component originated in the core and its temporal evolution we set $L=13$, since beyond this spatial resolution the magnetization of Earth’s crust makes it difficult to separate the core and crustal contributions to the interior magnetic field.

For the years before 2020 we describe the geomagnetic field via the IGRF-13 model^23^, which contains a description of the Gauss coefficients from 1900 to 2020, at 5 years of interval, and a forecast that is distributed for use between 2020 and 2025. For the years after 2020, we consider two state-of-the-art forecasts which we refer to by the principal home institution of the authors: one is presented in^31^ and we refer to it as the MPG forecast, and the other is presented in^30^, referred to as the IPGP forecast. Both MPG and IPGP estimations are based on convection-driven dynamo simulations using the PARODY code^56,57^. The dynamical MPG forecast is based on an ensemble Kalman filter approach, described in^58^, combined with localization techniques that allowed the authors to obtained converged results with a limited number of ensemble members. The background dynamo model used by the MPG forecast is of moderate complexity, with homogeneous heat flow boundary conditions. The observations assimilated consisted of the main field from the COV_OBS.x1 model^59^ for the epochs between 1840 and 2000 and from the Kalmag model^60^ for the epochs between 2001 and 2020. The MPG forecast, which starts in 2020, was a candidate model for the IGRF-13^23^. Together with the mean MPG forecast (obtained by averaging the coefficients of the 256 ensemble members) we consider 64 random members of the ensemble. This reduced ensemble size has been chosen as a compromise between the number of additional forecasts (and thus the computational complexity of the conversion between geographic and AACGM coordinates) and the capability of the reduced ensemble to meaningfully capture the spread of the total ensemble (see^31^).

The IPGP forecast is based on a single-epoch inversion, for which the initial condition of the forecast is estimated from main field and secular variation (SV) data for the 2015 epoch from the CHAOS-5 model^61^. As background state for the inversion and to evolve the initial condition forward in time, the IPGP forecast makes use of the Coupled Earth dynamo model^62^, which employs a more involved physical setup (such as gravitational coupling between the inner core and the mantle as well as heterogeneous mass anomaly and heat flow conditions at inner and outer boundaries, respectively) that helps better explain the observed field. Since the IPGP forecast starts in 2015 there is a potentially severe discontinuity between the IPGP and the IGRF-13 models for the years between 2015 and 2020. However, for the purposes of the present paper we could not find any significant discrepancies between the two (with the exception of the $g_1^1$ coefficient, see below), and we continued to adopt the 2020 epoch in the IGRF-13 model as the present, effectively discarding the first 5 years of the IPGP forecast. Both MPG and IPGP forecast strategies were tested through hindcast experiments^30,31^, showing good potential for magnetic field prediction over decadal time scales.

For comparison, we consider a third, simple forecast given by a linear extrapolation of the IGRF-13 coefficients past its epoch of validity according to the IGRF consortium (i.e., 2025). We acknowledge that extrapolation past the 2025 epoch is beyond the scope and purposes of the IGRF model. This forecast has no particular physical meaning and merely serves to compare the MPG and IPGP forecast with future scenarios derived with no consideration of the non-linear dynamics of the geodynamo. We refer to this forecast as the IGRF forecast.

In Supplementary Fig. S2 we compare a selection of model coefficients evolution for the three models and forecasts. In Supplementary Fig. S2a we show the past evolution and forecasts for the dipole intensity and for the geomagnetic North Pole latitude, defined respectively as:

3 $$\begin{aligned} m_1= & {} \sqrt{\left( g_1^0\right) ^2 + \left( g_1^1\right) ^2 + \left( h_1^1\right) ^2}, \end{aligned}$$

4 $$\begin{aligned} \uplambda _N= & {} \frac{180^\circ }{\pi }\text {arccos}\left( \frac{g_1^0}{m_1}\right) -90^\circ . \end{aligned}$$

In Supplementary Fig. S2b we show the change of the $l=1$ coefficients and dipolar intensity $m_1$ relative to their 2020 values. This is done in order to highlight the temporal variations, which would not be visible in the timeseries of the unmodified coefficients, given the large differences in the numerical values of the coefficients. In Supplementary Figures S2c and S2d we plot the unmodified $l=2$ and a selection of the $l=3$ Gauss coefficients. In these Figures, the root-mean-squared intensity $m_l$ is a generalisation of the definition (3):

5 $$\begin{aligned} m_l = \sqrt{\sum _{m=0}^l\left[ \left( g_l^m\right) ^2 + \left( h_l^m\right) ^2\right] }. \end{aligned}$$

Supplementary Fig. S2 shows that there can be significant variations in the evolution of the Gauss coefficients among the various forecasts. This is true even if we only consider the dipole components in the MPG and IPGP, both based on a dynamical description of the dynamics of Earth’s core. The dipole latitude and $g_1^0$ in the MPG and IPGP forecasts are close to each other, in agreement with the validation experiments illustrated in^30,31^, as are the forecasts of $h_1^1$. The MPG and IPGP $g_1^1$ forecast however show significant differences in the variations of this coefficient in the two models. For subsequent degrees *l* we can see either agreements or significant departures, depending on the specific coefficient we consider. For most of the coefficients and quantities displayed in Supplementary Fig. S2, the IGRF forecast, based on a linear extrapolation, gives the most extreme variations: see for example the dipole latitude in Supplementary Fig. S2a and $m_2$ in Supplementary Fig. S2c.

Although we made use of the set of Gauss coefficients up to degree $L=13$ contained in the field models described above, we find that, visually, the final shape of the auroral and danger zones is recovered for $L\le 5$.

## Supplementary Information

Supplementary Figures.

## Data Availability

The IPGP geomagnetic field forecast dataset, analyzed during the current study, is freely available at http://www.ipgp.fr/~aubert/tools.html#data. The MPG geomagnetic field forecast dataset, analyzed during the current study, is freely available at https://edmond.mpdl.mpg.de/dataset.xhtml?persistentId=doi:10.17617/3.MZGFPM. The IGRF and *gufm1* geomagnetic field models, analyzed during the current study, are both freely available as part of the aacgmv2 Python package (version 2.6.2) at: https://github.com/aburrell/aacgmv2. The auroral and danger zones boundary datasets, as well as the geomagnetic field forecasts and field models generated and analysed during the current study are available in the auroral_forecast repository: https://github.com/smaffei/auroral_forecast. The dataset for the aurorae sightings during the Carrington event displaied in Figure 2 have been provided by James L. Green and Scott Boardsen (NASA), and it is available from NASA upon reasonable request. The worldcities dataset, used in the generation of figures presented in this study, is freely available at: https://gitlab.huma-num.fr/nlambert/resources/-/tree/master/datasets/simplemaps_worldcities_basicv1.73.
