# Supplementary material for: Climatological predictions of the auroral zone locations driven by moderate and severe space weather events
Source: Sci Rep. 2023 Jan 15;13:779. doi: 10.1038/s41598-022-25704-2 (PMC9841014; doi:10.1038/s41598-022-25704-2)
Supplement: Supplementary file 1 — Supplementary Figures. [file 41598_2022_25704_MOESM1_ESM.pdf]

Climatological Predictions of the Auroral Zone Locations Driven by Moderate and Severe Space Weather Events: Supplementary Information

Stefano Maffei*[1,2], Joseph W. B. Eggington[3], Philip W. Livermore[2], Jonathan E. Mound[2], Sabrina Sanchez[4], Jonathan P. Eastwood[3] & Mervyn P. Freeman[5]

[1]Earth and Planetary Magnetism Group, Institute of Geophysics, ETH Zurich, Zürich, Switzerland.

[2]School of Earth and Environment, University of Leeds, Leeds, UK.

[3]Space and Atmospheric Physics Group, Blackett Laboratory, Imperial College London, London, UK

[4]Institut de Physique du Globe de Paris, Université Paris-Diderot, Paris, France

[5]British Antarctic Survey, High Cross, Madingley Road, Cambridge, UK

November 21, 2022

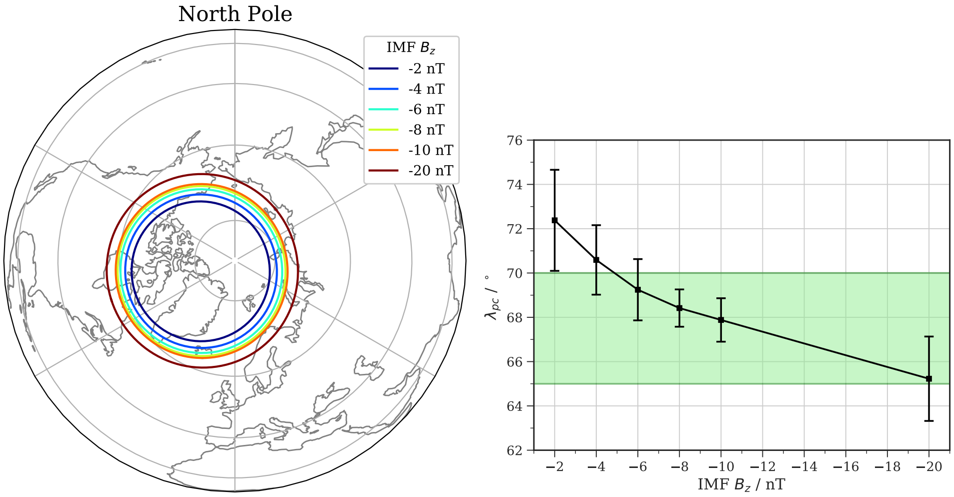

(a) (b)

Fig. S1: **Location of the northern border of the auroral zone from magnetospheric simulations.** Results from the MHD simulations for the ionospheric polar cap location under different strengths of IMF B_z driving. Shown in (a) is the result of transforming the mean polar cap latitudes from geomagnetic to geographic coordinates. This plot was generated in `Python 3.8` with the library `cartopy` (version 0.18.0). Plotted in (b) are the mean polar cap latitudes λ_{pc}, with the error bars showing the standard deviation along the open-closed field line boundary. The green band highlights the approximate geomagnetic latitudes defined as within the typical auroral zone in this study.

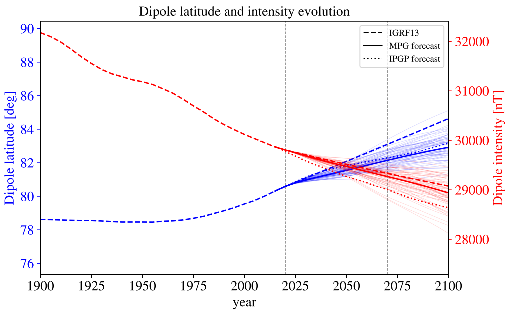

(a)

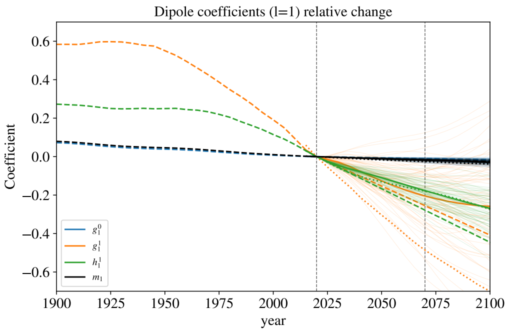

(b)

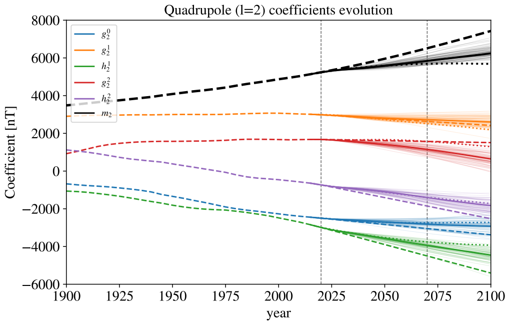

(c)

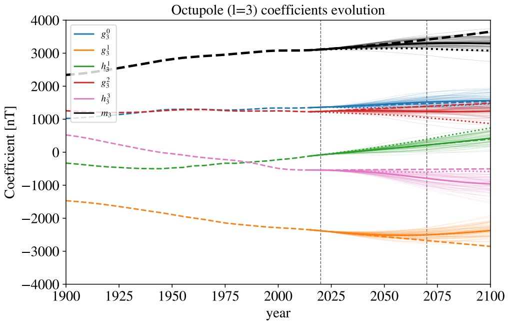

(d)

Fig. S2: **Comparison of the Gauss coefficients' temporal evolution as described by the models and forecasts used in this study.** Dashed lines refer to the IGRF-13 model (up to 2020) and forecast (after 2020); continuous thick and thin lines indicate, respectively, the mean MPG forecast and its esnemble members; dotted lines show the IPGP forecasts. The vertical dashed lines indicated the 2020 and 2070 epochs. The panels show the temporal evolution of: (a) the dipole intensity and latitude (see main text for definition); (b) the change in the $l = 1$ coefficients relative to their 2020 values; (c) the $l = 2$ coefficients; (d) selected $l = 3$ coefficients.

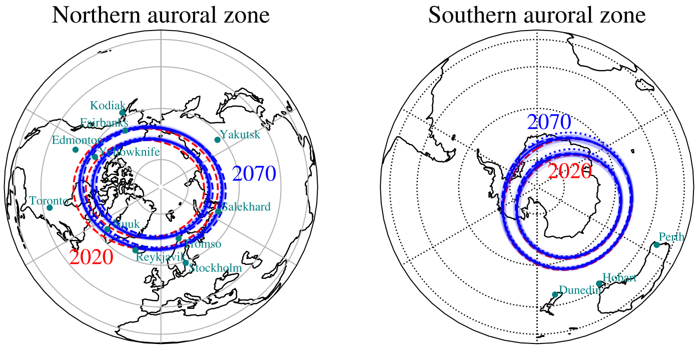

(a)

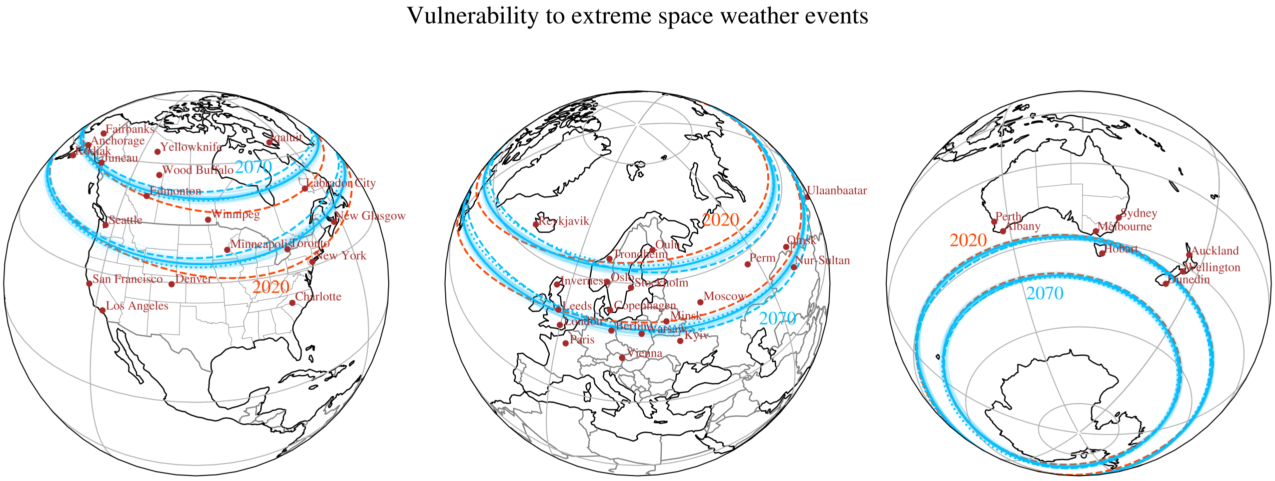

(b)

Fig. S3: **Auroral zones and danger zones for 2020 and 2070.** (a) Auroral zones poleward and equatorward boundaries for the 2020 (in red) and 2070 (in blue) epochs. The AACGM latitudes for the 2020 epoch are calculated from geomagnetic field coefficients given by the IGRF-13. For the 2070 epoch the AACGM coordinates where calculated from the IPGP forecast. (b) Poleward and equatorward boundaries of the regions exposed to severe space weather events ('danger zones') for the 2020 (orange) and 2070 (cyan) epochs (shown via a near-side projection). The AACGM latitudes for the 2020 epoch are calculated from geomagnetic field coefficients given by the IGRF-13. For the 2070 epoch the AACGM coordinates were calculated from the IGRF forecast (dashed lines), the MPG mean and ensemble members forecast (continuous thick and thin lines, respectively) and the IPGP forecast (dotted lines). These plots were generated in `Python 3.8` with the library `cartopy` (version 0.18.0).

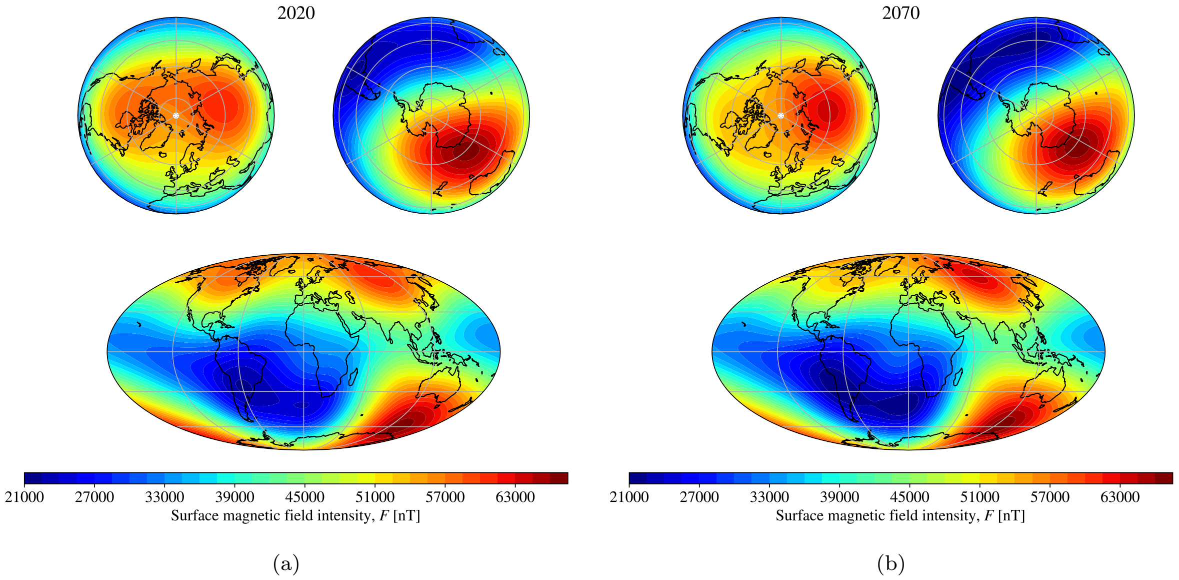

Fig. S4: **Geomagnetic field intensity at Earth's surface for 2020 and 2070.** Panels (a) and (b) show the geomagnetic field intensity for, respectively, the 2020 epoch (calculated from the IGRF-13 model) and the 2070 epoch (estimated from the MPG forecast). In both panels polar views are shown on top for both the Northern (left) and Southern (right) hemispheres and a global view is shown in the bottom via a Mollweide projection centered at 0° longitude. These plots were generated in `Python 3.8` with the library `cartopy` (version 0.18.0).
